# Time-Dependent Association of Thoracic Anthropometric Parameters with Survival After In-Hospital Cardiac Arrest: A Retrospective Single-Center Observational Study

**DOI:** 10.3390/jcm15134978

**Published:** 2026-06-26

**Authors:** Yong Oh Kim, Joonghyun Ahn, Jeong-Am Ryu

**Affiliations:** 1 Department of Emergency Medicine, Dankook University Hospital, School of Medicine, Dankook University, Cheonan 31116, Republic of Korea; ggggmmmmaaaail@gmail.com; 2 Biostatistics and Clinical Epidemiology Center, Samsung Medical Center, Seoul 06351, Republic of Korea; jhguy.ahn@samsung.com; 3 Department of Critical Care Medicine, Samsung Medical Center, School of Medicine, Sungkyunkwan University, Seoul 06351, Republic of Korea; 4 Department of Neurosurgery, Samsung Medical Center, School of Medicine, Sungkyunkwan University, Seoul 06351, Republic of Korea

**Keywords:** cardiopulmonary resuscitation, body mass index, chest computed tomography, time-dependent effects, personalized medicine, in-hospital cardiac arrest

## Abstract

**Background**: Current cardiopulmonary resuscitation (CPR) guidelines recommend a uniform chest compression depth (5–6 cm) for all adults, disregarding anatomical variability. The primary objective was to determine whether thoracic anthropometric parameters are associated with 28-day mortality after in-hospital cardiac arrest (IHCA); the secondary objective was whether these associations vary with CPR duration. **Methods**: In this retrospective single-center cohort, 431 adults with IHCA and available chest computed tomography (CT) were analyzed. Body mass index (BMI), internal anteroposterior diameter (IAPD), and external anteroposterior diameter (EAPD) were measured. Patients were stratified by CPR duration (≤5, 5–10, >10 min), and multivariable logistic regression with interaction terms tested time-dependent effects on 28-day mortality. **Results**: Overall 28-day survival was 40.8% (176/431). During the early phase (≤5 min), higher BMI, IAPD, and EAPD were each associated with increased mortality, and underweight patients had lower mortality than normal-weight and overweight patients. These anatomical associations attenuated and lost significance during prolonged resuscitation (>10 min), when CPR duration dominated outcomes. **Conclusions**: The prognostic value of body composition after IHCA is time-dependent, being greatest during the first five minutes, supporting individualized, body composition-guided chest compression—particularly using readily available BMI—during early resuscitation.

## 1. Introduction

Chest compression is the cornerstone of cardiopulmonary resuscitation (CPR), directly determining patient survival through maintenance of cardiac output and oxygen delivery to vital organs [1,2,3,4]. Since the 2015 American Heart Association Guidelines, a uniform compression depth of 5–6 cm has been recommended for all adults during CPR, representing a “one-size-fits-all” approach that has remained unchanged for nearly a decade [2]. This uniform 5–6 cm recommendation has been maintained in contemporary guidelines, including the 2020 American Heart Association Guidelines for CPR and Emergency Cardiovascular Care and the 2021 European Resuscitation Council Guidelines [5,6,7].

However, this standardized approach fundamentally ignores the substantial anatomical variability among adult patients. The anteroposterior chest diameter varies significantly across individuals, influenced by body mass index (BMI), age, sex, and underlying pathology [8,9,10,11,12]. While inadequate compression depth (<5 cm) reduces cardiac output and coronary perfusion pressure, excessive compression (>6 cm) increases the risk of mechanical complications including rib fractures and organ injury [8,11]. This creates a clinical paradox: the same compression depth may be simultaneously insufficient for larger patients and excessive for smaller patients.

Recent studies have attempted to address this limitation by proposing individualized compression depths based on chest computed tomography (CT) measurements [9,10,11,12]. However, these investigations have largely treated anatomical optimization as a static concept, assuming that personalized approaches remain equally beneficial throughout the entire resuscitation process. This assumption overlooks a critical dimension of CPR physiology: the temporal dynamics of cardiac arrest and resuscitation. The concept of “golden time” in emergency medicine suggests that therapeutic interventions may have time-dependent efficacy, with early interventions yielding disproportionately greater benefits than later ones [13]. In the context of CPR, this temporal relationship is particularly pronounced: survival from witnessed cardiac arrest decreases by 7–10% for every minute without intervention [14], and recent evidence demonstrates that delays in bystander CPR initiation are associated with progressively lower survival, with CPR initiated 10 min or later showing substantially reduced effectiveness [15]. This exponential decline in survival rates raises the fundamental question: does anatomical personalization maintain its clinical relevance throughout the entire duration of CPR, or does time eventually overwhelm individual anatomical advantages?

Current evidence lacks investigation into the interaction between anatomical factors and resuscitation duration. While previous studies have demonstrated that chest dimensions correlate with optimal compression depth [9,10,11,12], none have examined whether these relationships persist across different phases of CPR. Understanding this temporal dimension is crucial for developing evidence-based protocols that optimize resource allocation and clinical decision-making during cardiac arrest management. Therefore, this study aimed to move beyond the traditional “one-size-fits-all” paradigm by investigating the time-dependent effects of individual anatomical characteristics on CPR outcomes. Specifically, we examined whether the relationships between body composition metrics (BMI, internal anteroposterior diameter [IAPD], and external anteroposterior diameter [EAPD]) and survival vary according to CPR duration in patients with in-hospital cardiac arrest (IHCA). Our hypothesis was that anatomical personalization would demonstrate maximum clinical benefit during early resuscitation but diminish in importance as CPR duration increases, supporting a novel “time-dependent personalization” approach to cardiac arrest management. Accordingly, the primary objective of this study was to evaluate the association between thoracic anthropometric parameters and 28-day mortality after IHCA, and the secondary objective was to test whether these associations are time-dependent—maximal during early resuscitation and attenuating as CPR duration increases.

## 2. Materials and Methods

### 2.1. Study Population

**Figure 1:**
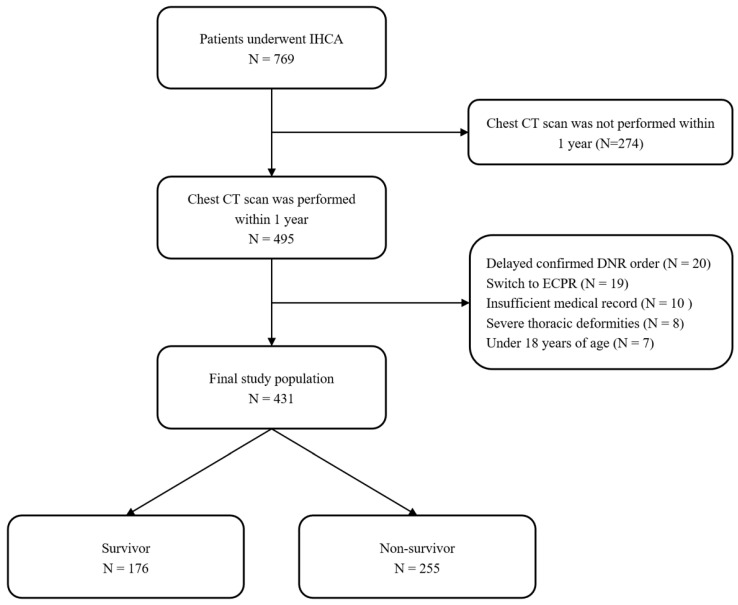
Study flow chart. IHCA, in-hospital cardiac arrest; CT, computed tomography; DNR, do-not-resuscitate; ECPR, extracorporeal cardiopulmonary resuscitation.

This was a retrospective, single-center, observational study designed to investigate the time-dependent effects of individual anatomical characteristics on CPR outcomes in adult patients who experienced IHCA between July 2016 and June 2019. This study was approved by the Institutional Review Board of Samsung Medical Center (IRB no. SMC 2020-06-170-001). The requirement for informed consent was waived due to its retrospective nature. Clinical and laboratory data were collected by trained study coordinators using a standardized case report form, with particular emphasis on precise timing of CPR initiation, duration, and anatomical measurements. Inclusion criteria were: (1) adult patients (≥18 years of age) who underwent CPR for IHCA during the study period, and (2) availability of chest CT scans performed within 1 year before cardiac arrest to enable measurement of IAPD and EAPD of the chest. Exclusion criteria were: (1) patients under 18 years of age, (2) those with severe thoracic deformities such as funnel chest, severe scoliosis or kyphosis, trauma-induced deformities, or cardiopulmonary structural abnormalities that would preclude accurate anatomical measurement or standard CPR technique, (3) those who discontinued resuscitation due to delayed confirmed do-not-resuscitate orders, (4) those who underwent extracorporeal CPR, which represents a fundamentally different resuscitation approach, and (5) those with insufficient medical records to determine precise CPR timing or anatomical measurements (Figure 1).

### 2.2. Definitions and Endpoints

**Figure 2:**
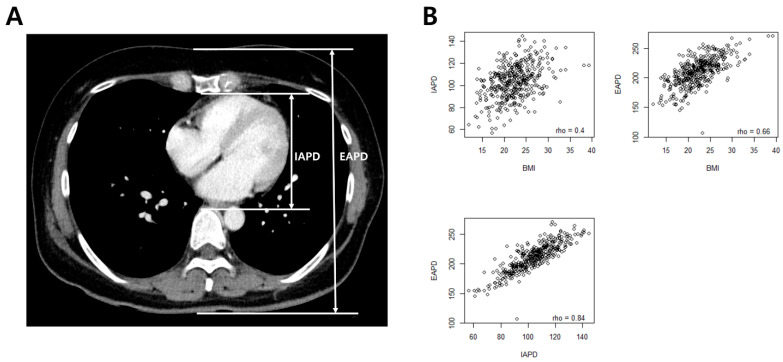
Computed tomographic scan demonstrating axial image at midpoint of the lower sternum which is a quarter of the total length of sternum and method for calculating external anteroposterior diameter (EAPD) and internal anteroposterior diameter (IAPD) of the chest (**A**). The relationship between body mass index (BMI) and IAPD (**B**). Scatter plots showing relationships between BMI and IAPD (Spearman’s rho = 0.40), BMI and EAPD (rho = 0.66), and IAPD and EAPD (rho = 0.84). All correlations were statistically significant (*p* < 0.001), demonstrating moderate to very strong intercorrelations among the anatomical measures. The particularly strong correlation between IAPD and EAPD (rho = 0.84) reflects their direct anatomical relationship as internal and external measurements of thoracic anteroposterior dimension.

The anteroposterior diameter of the thorax was measured by an axial slice at the lower half level of the sternum, the typical chest compression location, of the chest CT according to the 2015 American Heart Association Guidelines (Figure 2) [2,11]. The lower half of the sternum was defined as the midpoint of the lower sternum which is a quarter of the total length of sternum [11]. The IAPD of the chest was defined as the compressible diameter, representing the distance from the posterior side of sternum to the anterior vertebral body [9,11]. The EAPD of the chest was defined as the distance from the skin anteriorly to the skin posteriorly [8,9,11]. The value of BMI was calculated as kg/m^2^. We classified the selected subjects into underweight (<18.5 kg/m^2^), normal weight (≥18.5 to <25 kg/m^2^), and overweight (≥25 kg/m^2^) groups according to BMI with World Health Organization criteria. To investigate the central hypothesis of time-dependent anatomical effects, patients were stratified into three CPR duration groups based on the total duration of resuscitation efforts: ≤5 min, 5–10 min, and >10 min. This stratification was designed to capture the hypothesized temporal dynamics where anatomical factors would be most relevant during early CPR but become progressively less important as resuscitation duration increased. The 5-min threshold was chosen based on evidence that survival benefits from CPR diminish rapidly after this critical time window [15,16,17], with similar time-based stratification approaches used in previous cardiac arrest outcome studies [18,19,20]. All the CT studies were performed using 64-channel scanners (Light Speed VCT, GE Healthcare, Milwaukee, WI, USA). Investigator who was blinded to clinical information evaluated each of the patients’ CT scans using commercial image-viewing software (Centricity RA1000 PACS Viewer, GE Healthcare, Milwaukee, WI, USA) [21]. The primary endpoint was 28-day mortality, chosen to capture both immediate resuscitation success and short-term survival outcomes that might be influenced by the quality and appropriateness of initial CPR efforts.

### 2.3. Procedure

CPR was performed according to standard protocols by the hospital’s CPR team, consisting of medical or surgical residents, interns, nurses, a respiratory therapist, and a team leader (either a fellow or attending physician from the intensive care unit or emergency department). All team members completed Basic Life Support Provider or Advanced Cardiovascular Life Support Provider courses. Importantly, all CPR efforts followed the current “one-size-fits-all” approach with uniform chest compression depth of 5–6 cm and rate of 100–120 compressions per minute, regardless of individual patient anatomy [2]. This standardized approach provided the foundation for investigating whether anatomical variations in patient chest dimensions affected outcomes despite uniform compression depths. All resuscitation events were recorded by bedside nurses according to Utstein-style guidelines [22], with particular attention to precise timing of CPR initiation and duration to enable the time-stratified analysis central to this study’s hypothesis. The team leader monitored CPR performance including chest compression depth, rate, hands-off time, ventilation, and defibrillation according to established protocols [2]. Post-resuscitation care included targeted temperature management, percutaneous coronary intervention, coronary artery bypass grafting, or other interventions as clinically indicated [23]. Surface cooling and targeted temperature protocols were determined by intensive care unit physicians according to established institutional guidelines [24]. Resuscitation medications administered during CPR—including epinephrine, amiodarone, atropine, sodium bicarbonate, and calcium gluconate—and the number of defibrillation attempts were recorded for each event according to Utstein-style guidelines.

### 2.4. Statistical Analyses

All data are presented as means ± standard deviations for continuous variables and numbers (percentages) for categorical variables. Group differences were assessed using Student’s *t*-test for continuous variables and Chi-square test or Fisher’s exact test for categorical variables. Multivariable logistic regression analysis was performed to identify independent predictors of 28-day mortality. Covariates were selected based on established clinical relevance, including sex, Glasgow Coma Scale before arrest, first monitored cardiac rhythm (shockable vs. non-shockable), and malignancy. These variables are well-recognized prognostic factors in cardiac arrest outcomes and were included regardless of univariate significance levels. Spearman’s rank correlation coefficients were calculated to assess intercorrelations among body composition metrics (BMI, IAPD, EAPD). Since these anatomical variables showed substantial correlations, each metric was evaluated in a separate multivariable model to avoid multicollinearity and ensure stable coefficient estimates. To explore whether the effects of BMI, IAPD, and EAPD on mortality varied according to CPR duration, we constructed three interaction models: BMI × CPR duration, IAPD × CPR duration, and EAPD × CPR duration. Prior to fitting the interaction models, we used restricted cubic spline functions to visually examine potential non-linear relationships between CPR duration and each anatomical metric, as well as their associations with mortality. These exploratory spline curves indicated approximately linear relationships across the observed data range, supporting the use of standard interaction models. Based on the interaction analyses, we additionally performed time-stratified models to quantify effect sizes across clinically meaningful CPR duration phases: ≤5 min (golden time), 5–10 min (transition period), and >10 min (late phase). For each stratum, adjusted odds ratios and 95% confidence intervals were estimated for BMI (continuous and categorical: underweight < 18.5 kg/m^2^, normal weight 18.5–25 kg/m^2^, overweight ≥ 25 kg/m^2^), IAPD, and EAPD, adjusting for the same clinically relevant covariates. Statistical significance was defined as *p* < 0.05 (two-sided). All analyses were conducted using R Statistical Software (version 4.5.2; R Foundation for Statistical Computing, Vienna, Austria). In sensitivity analyses, the multivariable models were additionally adjusted for resuscitation interventions (epinephrine and sodium bicarbonate administration) to assess whether the associations between body composition and mortality were independent of intra-arrest emergency care.

## 3. Results

### 3.1. Study Population and Baseline Characteristics

A total of 431 patients who underwent IHCA with available chest CT scans were included in the final analysis (Figure 1). The overall 28-day survival rate was 40.8% (176/431). The mean patient age was 63.3 ± 14.4 years, and 273 (63.3%) patients were male. Baseline characteristics stratified by survival status are presented in Table 1. Malignancy was the most common comorbidity (67.5%), followed by hypertension (52.9%). Non-survivors had a significantly higher prevalence of malignancy compared to survivors (73.7% vs. 58.5%, *p* = 0.001). Survivors had a higher Glasgow Coma Scale before arrest (13.4 ± 3.0 vs. 12.1 ± 4.1, *p* < 0.001) and were more likely to have a shockable initial rhythm (26.7% vs. 12.2%, *p* < 0.001). The most critical difference between groups was CPR duration, with non-survivors experiencing significantly longer resuscitation attempts (15.9 ± 14.9 min vs. 6.0 ± 6.7 min, *p* < 0.001). Post-cardiac arrest care including targeted temperature management was performed in 11 (2.6%) patients. Non-survivors received epinephrine (89.4% vs. 65.9%), sodium bicarbonate (35.7% vs. 8.5%), and calcium gluconate (28.6% vs. 5.7%) significantly more often than survivors (all *p* < 0.001), with a higher cumulative epinephrine dose (median 5 vs. 1 ampoules, *p* < 0.001), whereas defibrillation frequency was similar between groups (Table 1). In sensitivity analyses additionally adjusting for epinephrine and sodium bicarbonate administration, the time-dependent associations between thoracic anatomical parameters and 28-day mortality were preserved, with anatomical metrics associated with mortality during early but not prolonged resuscitation.

### 3.2. Body Composition Metrics and Time-Stratified Analysis

Body composition metrics stratified by CPR duration and survival status are shown in Table 2. Overall, survivors had significantly lower BMI compared to non-survivors (21.8 ± 4.5 kg/m^2^ vs. 22.7 ± 3.6 kg/m^2^, *p* = 0.028), with BMI category distribution also differing significantly between groups (*p* = 0.003). Chest dimensions (IAPD and EAPD) showed non-significant trends toward larger values in non-survivors (*p* = 0.111 and 0.110, respectively). Time-stratified analysis revealed marked temporal variation in the prognostic significance of body composition metrics. During the early phase (CPR ≤ 5 min), BMI was significantly associated with mortality (*p* = 0.025), with underweight patients showing the lowest mortality risk (18.8%) compared to normal weight (43.8%) and overweight (52.2%) groups. BMI categories demonstrated strong discrimination during this golden time period (*p* = 0.010). In contrast, during the late phase (>10 min), body composition metrics completely lost their prognostic value, with no significant differences observed for BMI (*p* = 0.700), BMI categories (*p* = 0.200), IAPD (*p* = 0.830), or EAPD (*p* = 0.847). This time-dependent attenuation underscores that anatomical factors exert their strongest influence during the initial critical minutes of resuscitation, with prognostic significance diminishing as CPR duration extends beyond 5 min. The relationship between BMI and IAPD is illustrated in Figure 2. Body composition metrics demonstrated moderate to strong intercorrelations, as anticipated given their shared anatomical basis. BMI showed a moderate positive correlation with IAPD (Spearman’s rho = 0.40, *p* < 0.001) and a strong positive correlation with EAPD (rho = 0.66, *p* < 0.001). IAPD and EAPD were very strongly correlated with each other (rho = 0.84, *p* < 0.001), reflecting the proportional relationship between internal and external thoracic dimensions.

### 3.3. Time-Dependent Associations Between Body Composition and Mortality

In multivariable logistic regression analysis adjusting for clinically relevant covariates, several factors were independently associated with 28-day mortality. Among baseline characteristics, the presence of malignancy (adjusted OR 2.03, 95% CI 1.23–3.35, *p* = 0.006) and lower Glasgow Coma Scale score before arrest (adjusted OR 0.88 per point, 95% CI 0.82–0.94, *p* < 0.001) were significant predictors of increased mortality. Initial cardiac rhythm also influenced outcomes, with shockable rhythm (ventricular tachycardia or ventricular fibrillation) associated with improved survival (adjusted OR 0.43, 95% CI 0.20–0.92, *p* = 0.029).

To investigate whether the effects of body composition metrics on mortality varied by resuscitation duration, we tested interaction terms between each anatomical measure and CPR duration in separate multivariable models (Model 1: BMI × CPR duration, Model 2: IAPD × CPR duration, Model 3: EAPD × CPR duration). Significant interactions were identified for IAPD (*p* = 0.038) and EAPD (*p* = 0.038), with a trend toward significance for BMI (*p* = 0.067), indicating time-dependent effects of these anatomical factors on mortality. Based on these interaction models, we performed time-stratified analyses across three CPR duration phases to quantify the magnitude and direction of anatomical effects at different resuscitation time points (Table 3). During the golden time (≤5 min), all anatomical metrics demonstrated significant associations with mortality. Higher BMI (adjusted OR 1.15 per kg/m^2^, 95% CI 1.05–1.26, *p* = 0.003), larger IAPD (adjusted OR 1.029 per cm, 95% CI 1.010–1.049, *p* = 0.003), and larger EAPD (adjusted OR 1.014 per cm, 95% CI 1.004–1.024, *p* = 0.008) were associated with increased mortality. When BMI was analyzed categorically with underweight patients as the reference group, both normal weight (adjusted OR 3.44, 95% CI 1.42–8.35, *p* = 0.007) and overweight (adjusted OR 2.91, 95% CI 1.11–7.64, *p* = 0.030) categories showed significantly higher mortality during this early phase.

In striking contrast, during the transition period (5–10 min) and late phase (>10 min), none of the anatomical metrics showed statistically significant associations with mortality (all *p* > 0.05). For instance, the effect of BMI shifted from adjusted OR 1.15 (*p* = 0.003) during the golden time to OR 1.04 (*p* = 0.489) during the transition period and adjusted OR 0.97 (*p* = 0.584) during the late phase. Similarly, the effects of IAPD and EAPD diminished over time, becoming non-significant in prolonged resuscitation scenarios. This time-dependent attenuation of anatomical effects demonstrates that body composition characteristics are most clinically relevant during the initial resuscitation period, with their prognostic value diminishing as CPR duration extends beyond 5 min.

**Figure 3:**
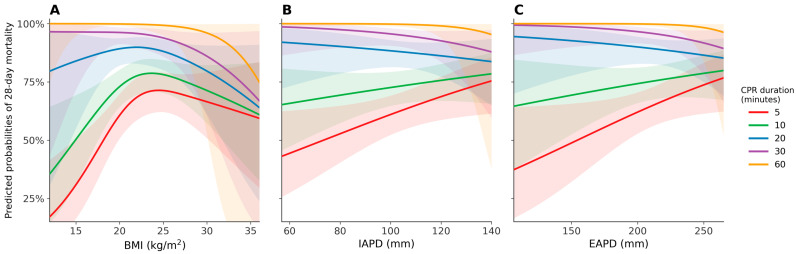
Time-dependent relationships between body composition metrics and 28-day mortality during cardiopulmonary resuscitation (CPR). Predicted probabilities from restricted cubic spline models show (**A**) U-shaped body mass index (BMI) effect with normal weight as highest risk group (obesity paradox), (**B**) positive internal anteroposterior diameter (IAPD)-mortality association, and (**C**) positive external anteroposterior diameter (EAPD)-mortality association. All anatomical effects are strongest during early CPR (red lines, 5 min) and diminish with prolonged resuscitation. Lines represent point estimates with 95% confidence intervals (shaded areas). Interaction *p*-values: BMI × CPR duration = 0.067, EAPD × CPR duration = 0.038, IAPD × CPR duration = 0.038.

Figure 3 illustrates the time-dependent relationships between body composition metrics and 28-day mortality using restricted cubic spline models. Panel A demonstrates the U-shaped BMI effect, with normal weight patients showing the highest mortality risk during early CPR (red lines), consistent with the obesity paradox phenomenon. Panels B and C show positive associations between chest dimensions (IAPD and EAPD) and mortality risk, with the strongest effects observed during the first 5 min of resuscitation. The progressive flattening of curves from red (5 min) to orange (60 min) visually represents the diminishing impact of anatomical factors over time. This graphical evidence supports the statistical finding that personalized approaches based on body composition are most beneficial during the critical “golden time” window.

Figure 3 illustrates the time-dependent relationships between body composition metrics and 28-day mortality using restricted cubic spline models. Panel A demonstrates a U-shaped relationship between BMI and mortality, with normal weight patients showing higher mortality risk during early CPR (red lines) compared to underweight and overweight patients. Panels B and C show positive associations between chest dimensions (IAPD and EAPD) and mortality risk, with the strongest effects observed during the first 5 min of resuscitation. The progressive flattening of curves from red (≤5 min) through yellow (5–10 min) to orange (>10 min) visually demonstrates the diminishing impact of body composition metrics as CPR duration increases, consistent with the significant interaction terms observed in multivariable models.

Collectively, these time-stratified analyses demonstrate that the prognostic impact of body composition on cardiac arrest outcomes is confined to the early resuscitation phase. During the first 5 min of CPR, marked disparities in survival were observed across body composition categories under current compression practices, with underweight patients demonstrating substantially lower mortality (18.8%) compared to normal weight (43.8%) and overweight (52.2%) patients (*p* = 0.010). These differential outcomes across anatomical phenotypes suggest that a uniform compression depth approach may not be equally effective for all patients, particularly during the critical early phase when body composition exerts its strongest prognostic influence. After 10 min of CPR, these anatomical effects disappeared entirely, with CPR duration becoming the dominant prognostic factor.

## 4. Discussion

This study investigated whether the effects of body composition on cardiac arrest outcomes vary by CPR duration, challenging the current “one-size-fits-all” approach to chest compression depth. In 431 patients with in-hospital cardiac arrest, we demonstrated three principal findings. First, survivors had significantly lower BMI and a higher proportion of underweight patients compared to non-survivors. Second, time-stratified analysis revealed that body composition metrics (BMI, IAPD, EAPD) demonstrated strong prognostic significance during the early phase (CPR ≤ 5 min), with underweight patients showing markedly lower mortality compared to normal weight and overweight groups. Third, these anatomical effects progressively attenuated over time, becoming completely non-significant during prolonged resuscitation (>10 min), as confirmed by significant interaction terms between body composition and CPR duration in multivariable models. These findings suggest that personalized chest compression depth based on individual anatomical characteristics may be most beneficial during the initial critical minutes of resuscitation, with implications for refining current CPR guidelines.

Eventually, these findings suggest that the worse outcomes observed in normal and overweight patients during early CPR may reflect insufficient compression depth to overcome their greater chest wall thickness and achieve adequate cardiac compression. Our results support a body composition metrics-based strategy for optimizing chest compression depth during the critical first 5 min of resuscitation. Among the body composition metrics we evaluated, BMI offers the most immediate practical value as it is readily available at the bedside without requiring imaging. A practical approach would involve targeting deeper compressions (6–7 cm, approaching or exceeding the upper limit of current guidelines) in patients with BMI ≥ 18.5 kg/m^2^ during early CPR, while maintaining standard depths (5–6 cm) in underweight patients. For institutions with access to recent chest CT imaging, chest dimensions (IAPD and EAPD) may provide even more anatomically precise guidance for compression depth optimization, though this requires further validation. Importantly, after 10 min of CPR, when body composition effects disappear and CPR duration dominates outcomes, this personalized approach becomes less relevant, and efforts should focus on minimizing resuscitation time through rapid identification of reversible causes. This body composition-based strategy can be implemented immediately using BMI in any clinical setting, with potential refinement through CT-derived metrics when available.

High-quality CPR is important to maintain cardiac output and ensure blood supply to major organs, thereby improving survival rate and reducing irreversible cerebral injury from hypoxic ischemia [2,25]. Among the key components of high-quality CPR, adequate chest compression depth is particularly important [1,8,10,11]. Shallow compression depths fail to maintain adequate cardiac output and blood flow to vital organs [8]. Conversely, excessively deep chest compressions can cause various mechanical complications and intra-organ injuries, including flail chest resulting from rib, sternal, or clavicular fractures; lung injuries such as pneumothorax, hemothorax, lung contusion, or pneumomediastinum; or abdominal injuries such as liver laceration or spleen injury [8,26,27,28,29]. Therefore, tailoring compression depth to patients’ body size may be crucial for optimizing clinical outcomes. The importance of considering individual body size in relation to compression depth has been previously recognized in pediatric resuscitation, where body size increases substantially with age [9]. Similar considerations should apply to adult patients, given the substantial inter-individual variation in body composition.

However, our findings revealed that patients with smaller chest dimensions (lower IAPD and EAPD) demonstrated better survival during the early phase of CPR, particularly among underweight individuals who showed substantially lower mortality compared to normal weight and overweight groups. This counterintuitive finding may be explained by the relationship between chest anatomy and compression effectiveness. In patients with smaller thoracic dimensions, the current guideline-recommended compression depth of 5–6 cm may represent a relatively deeper penetration proportional to their chest size, potentially achieving more effective myocardial compression and coronary perfusion without causing excessive injury [1,30]. Moreover, the lower complication risk in these patients suggests that deeper compressions beyond the current recommendations might be not only safe but potentially beneficial for smaller individuals [31]. The chest wall compliance and compressibility in lean patients may allow for deeper compressions with adequate force generation while minimizing the risk of skeletal or visceral injuries [8,32].

Conversely, patients with larger chest dimensions (higher IAPD and EAPD) experienced worse outcomes during the early resuscitation phase when applying the standard compression depth. In these individuals, the uniform 5–6 cm compression may represent insufficient penetration relative to their thoracic size, failing to achieve adequate myocardial compression and coronary perfusion pressure [33,34]. IAPD serves as a clinically relevant indicator of thoracic size and represents the compressible anterior–posterior diameter, making it a useful parameter for estimating adequate chest compression depth [8,9,10,11]. The larger distance from the sternum to the heart in these patients means that the same absolute compression depth translates to less effective cardiac compression, potentially compromising hemodynamic efficacy during CPR [4,35]. This finding aligns with the principle that compression depth should be proportional to chest dimensions to ensure adequate blood flow generation [4,10]. Our results suggest that larger patients might benefit from deeper compressions exceeding current recommendations, personalized to their individual anatomical characteristics to optimize resuscitation outcomes.

A striking finding was the U-shaped relationship between BMI and early mortality. Underweight patients demonstrated the most favorable outcomes, while normal weight patients paradoxically experienced the highest mortality, and overweight patients showed intermediate mortality that was nonetheless substantially better than normal weight. Several mechanisms may explain these differential outcomes. For underweight patients, standard compression depth may achieve relatively deeper myocardial compression [30], with additional benefits from lower metabolic oxygen demand [36] and favorable chest wall mechanics [8,32]. The superior survival in overweight and obese patients compared to normal weight individuals aligns with the “obesity paradox” observed in cardiovascular conditions [37,38], potentially reflecting metabolic reserves or protective effects of adipose tissue during ischemia, though mechanisms in acute arrest may differ from chronic disease states. However, BMI limitations as a body composition measure—including inability to distinguish lean mass from adipose tissue or account for fat distribution—suggest cautious interpretation [39].

A critical insight from our study is that the prognostic significance of body composition metrics was confined predominantly to the early phase of resuscitation (≤5 min), with their effects diminishing progressively as CPR duration extended. CPR duration emerged as an overwhelmingly powerful predictor of outcomes, substantially eclipsing the effects of anatomical factors as resuscitation time progressed. The significant interaction terms between body composition metrics and CPR duration in our multivariable models demonstrate that the prognostic value of anatomical measures is not constant but rather diminishes progressively with time. This time-dependent attenuation can be explained by the overwhelming influence of CPR duration on outcomes [20,40]. During prolonged resuscitation, the cumulative effects of circulatory arrest—including progressive myocardial dysfunction, metabolic acidosis, electrolyte derangements, and multi-organ ischemic injury—become the dominant determinants of survival, overshadowing the relatively modest contributions of anatomical factors. In contrast, during the golden time when prompt restoration of circulation is achievable, optimal chest compression depth tailored to individual anatomy may critically influence the adequacy of coronary and cerebral perfusion thereby affecting the likelihood of successful resuscitation [7,40,41,42]. This temporal hierarchy of prognostic factors has important implications for understanding resuscitation pathophysiology. During brief resuscitation attempts when myocardial viability is preserved and the likelihood of return of spontaneous circulation is high, optimizing mechanical factors such as compression depth based on individual anatomy may meaningfully impact outcomes [43]. However, as CPR duration extends beyond 10 min, the cumulative burden of global ischemia becomes so profound that anatomical considerations become clinically irrelevant. This suggests that during prolonged resuscitation, the focus should shift from fine-tuning compression depth based on body habitus to addressing other critical determinants of survival, such as prompt identification and correction of reversible causes, maintenance of adequate oxygenation and ventilation, and consideration of advanced interventions such as extracorporeal CPR in appropriate candidates [16,44,45,46].

The quality of chest compressions encompasses not only depth but also rate and continuity. In pediatric in-hospital cardiac arrest, a chest compression rate of 80 to <100 per minute has been associated with improved survival to discharge and favorable neurological outcome [47], whereas longer chest compression pauses have been associated with a lower probability of return of spontaneous circulation [48]. Together, these findings underscore that the adequacy, rate, and uninterrupted delivery of compressions all influence resuscitation outcomes. Our finding that anatomically appropriate compression depth is most consequential during the early phase is consistent with this principle, as the early minutes are when high-quality, minimally interrupted compressions most strongly determine outcomes. Contemporary data indicate that survival to discharge after IHCA remains approximately 25%, with return of spontaneous circulation achieved in roughly half of events, and outcomes are strongly determined by resuscitation duration and arrest characteristics [49]. Our observation that CPR duration overwhelms anatomical factors during prolonged resuscitation is concordant with this literature and reinforces that anatomy-guided strategies are most applicable when early return of spontaneous circulation remains achievable.

Central obesity, reflected in our study by larger internal and external anteroposterior thoracic diameters, increases the chest-wall depth that a fixed 5–6 cm compression must overcome, so that the same absolute depth yields proportionally shallower cardiac compression in patients with greater central adiposity. This may explain the higher early mortality observed with larger IAPD/EAPD and higher BMI and is distinct from the chronic ‘obesity paradox’ described in cardiovascular disease; in the acute arrest setting, greater thoracic dimensions appear mechanically disadvantageous during standard-depth compressions.

Beyond compression mechanics, ventilation strategy during CPR is also under active investigation. Experimental and early clinical studies suggest that chest compression-synchronized ventilation (CCSV), in which short positive-pressure breaths are delivered synchronously with each compression, may improve arterial oxygenation and carbon dioxide elimination compared with intermittent positive-pressure ventilation (IPPV) [50]. Although our study did not address ventilation, these findings reinforce the overarching concept that resuscitation may benefit from individualized, physiology-guided optimization rather than a uniform approach.

This study has several limitations. First, the retrospective observational design limits causal inference and may introduce unmeasured confounding. Second, chest CT scans were not systematically obtained in all cardiac arrest patients but rather performed for other clinical indications, potentially introducing selection bias. Third, despite standardized CPR protocols with real-time feedback, actual compression depth may have varied due to rescuer factors, and granular compression data were not available. Fourth, as a single-center study at a tertiary academic medical center, generalizability to other settings, particularly community hospitals and out-of-hospital cardiac arrests, may be limited. Fifth, our modest sample size (*n* = 431) limited statistical power for subgroup analyses, resulting in wide confidence intervals for some estimates. Finally, the post-hoc time-stratified analysis requires prospective validation. Future multicenter randomized trials with real-time anatomical measurements and personalized compression depth targets are needed to establish whether individualized strategies improve outcomes. In addition, although resuscitation medications were administered more frequently in non-survivors and were strongly correlated with resuscitation duration, adjustment for these interventions did not alter the principal findings; nonetheless, residual confounding by intra-arrest emergency care cannot be fully excluded.

## 5. Conclusions

This study demonstrates that body composition effects on cardiac arrest outcomes are profoundly time-dependent, challenging the “one-size-fits-all” compression depth paradigm. During early CPR (≤5 min), anatomical metrics (BMI, IAPD, EAPD) strongly predicted mortality, with underweight patients showing markedly superior survival compared to normal and overweight groups. However, these effects disappeared beyond 10 min when CPR duration became the dominant factor. These findings support a body composition metrics-based approach to compression depth during early resuscitation. BMI, being immediately available at bedside, enables practical implementation—specifically, targeting deeper compressions approaching or exceeding current guideline upper limits for patients with higher BMI during the first 5 min. CT-derived metrics may offer additional precision when available. After 10 min, focus should shift to minimizing resuscitation duration. Prospective studies with real-time depth monitoring are needed to validate this individualized approach in clinical practice.

## Figures and Tables

**Table 1:** Baseline characteristics.

	Survivor (*n* = 176)	Non-Survivor (*n* = 255)	*p* Value
Age (yr)—mean ± SD	62.9 ± 13.6	63.7 ± 14.9	0.577
Gender, male—no. of patients (%)	120 (68.2)	153 (60.0)	0.103
Comorbidities—no. of patients (%)			
Malignancy	103 (58.5)	188 (73.7)	0.001
Hypertension	103 (58.5)	125 (49.0)	0.065
Diabetes mellitus	75 (42.6)	87 (34.1)	0.091
Previous myocardial infarction	46 (26.1)	42 (16.5)	0.020
Dyslipidemia	58 (33.0)	66 (25.9)	0.137
Chronic kidney disease ^a^	50 (28.4)	48 (18.8)	0.027
Chronic obstructive pulmonary disease	25 (14.2)	36 (14.1)	0.999
Chronic liver disease ^b^	13 (7.4)	30 (11.8)	0.184
Glasgow Coma Scale before arrest—mean ± SD	13.4 ± 3.0	12.1 ± 4.1	<0.001
Cardiac cause of arrest—no. of patients (%)			0.003
Ischemic	15 (8.5)	5 (2.0)	
Non-ischemic	161 (91.5)	250 (98.0)	
Location of CPR—no. of patients (%)			0.016
Intensive care unit	86 (48.9)	133 (52.2)	
General ward	49 (27.8)	57 (22.4)	
Emergency room	26 (14.8)	58 (22.7)	
Operation room	6 (3.4)	4 (1.6)	
Other	9 (5.1)	3 (1.2)	
First monitored rhythm—no. of patients (%)			<0.001
Asystole	33 (18.8)	66 (25.9)	
Pulseless electrical activity	83 (47.2)	152 (59.6)	
Shockable rhythm (VT or VF)	47 (26.7)	31 (12.2)	
Unknown	13 (7.4)	6 (2.4)	
Defibrillation—no. of patients (%)	42 (23.9)	55 (21.6)	0.657
Resuscitation medications—no. of patients (%)			
Epinephrine	116 (65.9)	228 (89.4)	<0.001
Amiodarone	7 (4.0)	21 (8.2)	0.118
Atropine	10 (5.7)	26 (10.2)	0.137
Sodium bicarbonate	15 (8.5)	91 (35.7)	<0.001
Calcium gluconate	10 (5.7)	73 (28.6)	<0.001
CPR duration (min)—mean ± SD	6.0 ± 6.7	15.9 ± 14.9	<0.001
Post-cardiac arrest management—no. of patients (%)			0.001
Target temperature management (surface cooling)—no. of patients (%)	6 (3.4)	5 (2.0)	
CAG or PCI—no. of patients (%)	15 (8.5)	4 (1.6)	

^a^ Chronic kidney disease is defined as either kidney damage or GFR < 60 mL/min/1.73 m^2^ for ≥3 months. ^b^ Chronic liver disease is defined as clinical evidence of cirrhosis, persistent liver dysfunction for ≥6 months, or imaging findings consistent with chronic liver pathology. SD, standard deviation; CPR, cardiopulmonary resuscitation; VT, ventricular tachycardia; VF, ventricular fibrillation; CAG, coronary angiography; PCI, percutaneous coronary intervention.

**Table 2:** Survival-associated body composition metrics across CPR duration strata.

Variables	Overall Survivor (*n* = 176)	Overall Non- Survivor (*n* = 255)	Overall *p*	CPR ≤ 5 min Survivor (*n* = 114)	CPR ≤ 5 min Non- Survivor (*n* = 82)	CPR ≤ 5 min *p*	CPR 5–10 min Survivor (*n* = 33)	CPR 5–10 min Non- Survivor (*n* = 45)	CPR 5–10 min *p*	CPR > 10 min Survivor (*n* = 29)	CPR > 10 min Non- Survivor (*n* = 128)	CPR > 10 min *p*
Age (yr)—mean ± SD	62.9 ± 13.6	63.7 ± 14.9	0.577	62.4 ± 13.4	61.7 ± 16.0	0.738	63.7 ± 14.1	63.1 ± 13.4	0.854	64.0 ± 14.4	65.2 ± 14.7	0.704
Gender, male—no. (%)	120 (68.2)	153 (60.0)	0.103	75 (65.8)	48 (58.5)	0.375	23 (69.7)	23 (51.1)	0.157	22 (75.9)	82 (64.1)	0.319
Body Composition Metrics												
BMI (kg/m^2^)—mean ± SD	21.8 ± 4.5	22.7 ± 3.6	0.028	21.6 ± 4.5	23.0 ± 3.7	0.025	21.8 ± 4.2	23.6 ± 4.2	0.073	22.4 ± 5.0	22.1 ± 3.3	0.700
BMI categories—no. (%)			0.003			0.010			0.157			0.200
Underweight (<18.5 kg/m^2^)	40 (23.3)	27 (11.0)		26 (23.4)	6 (7.6)		8 (24.2)	4 (8.9)		6 (21.4)	17 (14.0)	
Normal weight (18.5–25 kg/m^2^)	96 (55.8)	162 (66.1)		63 (56.8)	49 (62.0)		18 (54.5)	27 (60.0)		15 (53.6)	86 (71.1)	
Overweight (≥25 kg/m^2^)	36 (20.9)	56 (22.9)		22 (19.8)	24 (30.4)		7 (21.2)	14 (31.1)		7 (25.0)	18 (14.9)	
IAPD (mm)—mean ± SD	102.2 ± 15.7	104.6 ± 14.8	0.111	102.2 ± 16.5	106.3 ± 15.3	0.075	101.6 ± 15.9	104.0 ± 15.9	0.503	103.0 ± 12.1	103.6 ± 14.1	0.830
EAPD (mm)—mean ± SD	208.1 ± 24.1	211.8 ± 22.5	0.110	207.4 ± 24.8	211.8 ± 21.1	0.191	207.3 ± 23.8	213.3 ± 28.3	0.318	212.0 ± 22.2	211.2 ± 21.2	0.847
CPR Characteristics												
CPR duration (min)—mean ± SD	6.0 ± 6.7	15.9 ± 14.9	<0.001	2.6 ± 1.5	2.9 ± 1.3	0.115	7.4 ± 1.5	7.7 ± 1.4	0.409	17.9 ± 8.4	27.2 ± 13.5	0.001

BMI, body mass index; SD, standard deviation; IAPD, internal anteroposterior diameter; EAPD, external anteroposterior diameter; CPR, cardiopulmonary resuscitation.

**Table 3:** Time-stratified analysis of anatomical predictors on 28-day mortality.

CPR Duration/Variables	Adjusted OR (95% CI)	*p* Value
CPR ≤ 5 min (Golden Time)		
BMI (per kg/m^2^)	1.15 (1.05–1.26)	0.003
Underweight (<18.5 kg/m^2^)	1 (Reference)	Reference
Normal vs. underweight	3.44 (1.42–8.35)	0.007
Overweight vs. underweight	2.91 (1.11–7.64)	0.030
IAPD (per cm)	1.029 (1.010–1.049)	0.003
EAPD (per cm)	1.014 (1.004–1.024)	0.008
CPR 5–10 min (Transition Period)		
BMI (per kg/m^2^)	1.04 (0.93–1.16)	0.489
Underweight (<18.5 kg/m^2^)	1 (Reference)	Reference
Normal vs. underweight	1.89 (0.67–5.35)	0.231
Overweight vs. underweight	1.75 (0.59–5.21)	0.315
IAPD (per cm)	1.011 (0.995–1.028)	0.189
EAPD (per cm)	1.008 (0.998–1.018)	0.124
CPR > 10 min (Late Phase)		
BMI (per kg/m^2^)	0.97 (0.87–1.08)	0.584
Underweight (<18.5 kg/m^2^)	1 (Reference)	Reference
Normal vs. underweight	1.12 (0.45–2.78)	0.816
Overweight vs. underweight	1.34 (0.52–3.44)	0.542
IAPD (per cm)	1.001 (0.985–1.017)	0.924
EAPD (per cm)	1.002 (0.992–1.012)	0.671

BMI, body mass index; IAPD, internal anteroposterior diameter; EAPD, external anteroposterior diameter; CPR, cardiopulmonary resuscitation; CI, confidence interval; OR, odds ratio. All models adjusted for sex, Glasgow Coma Scale before arrest, pre-TIA/stroke, first monitored rhythm (shockable vs. non-shockable), and malignancy. Time stratification demonstrates differential effects of anatomical predictors across CPR duration phases.

## Data Availability

Regarding data availability, our data are available on the Harvard Dataverse Network (http://dx.doi.org/10.7910/DVN/2WR3IW).

## Abbreviations

BMI, body mass index; CI, confidence interval; CPR, cardiopulmonary resuscitation; CT, computed tomography; EAPD, external anteroposterior diameter; IAPD, internal anteroposterior diameter; IHCA, in-hospital cardiac arrest; OR, odd ratio.
